# Risk factors for low‐risk prostate cancer: A retrospective cohort study within the FinRSPC trial

**DOI:** 10.1002/ijc.70026

**Published:** 2025-07-17

**Authors:** Uzoamaka E. Okwor, Jani Raitanen, Kirsi Talala, Teuvo L. J. Tammela, Kimmo Taari, Paula Kujala, Anssi Auvinen

**Affiliations:** ^1^ Faculty of Social Sciences Tampere University Tampere Finland; ^2^ UKK Institute for Health Promote Research Tampere Finland; ^3^ Cancer Society of Finland Helsinki Finland; ^4^ Faculty of Medicine and Health Technology Tampere University Hospital, Department of Urology, and Tampere University Tampere Finland; ^5^ Medical Faculty Helsinki University Hospital, Department of Urology, and University of Helsinki Helsinki Finland; ^6^ FimLab Laboratories Pathology Service Tampere Finland

**Keywords:** cohort, overdiagnosis, prostate cancer, screening

## Abstract

Overdiagnosis of low‐risk prostate cancer (PC), often accompanied by overtreatment, remains an important harmful consequence of prostate‐specific antigen (PSA)‐based screening. Although PSA screening can reduce PC mortality and metastatic PC, the balance of benefits and harms remains controversial. This retrospective cohort study of 80,144 men from the Finnish Randomized Study of Screening for Prostate Cancer, with a median follow‐up of 18.0 years, compared determinants of low‐risk PC with determinants of high‐risk PC. Low‐risk PC (*N* = 1774) was classified according to the European Association of Urology guidelines, excluding cases with subsequent PC death. A secondary analysis excluded cases with post‐diagnosis disease progression. Intermediate, high‐risk, and advanced cases were classified as high‐risk PC (*N* = 6466). Poisson regression was used to analyze PC incidence. Low‐risk PC was more common in the screening than the control arm (1.9 vs. 1.2 cases per 1000 person‐years), whereas high‐risk PC was more frequent in the control arm (5.7 vs. 5.4 cases per 1000 person‐years in the screening arm). The risk of low‐risk PC remained stable across screening rounds, while the risk for high‐risk PC declined after the first screen. Age was associated with an increased risk of high‐risk PC, but no clear trend by age was observed for low‐risk PC. Family history and use of 5‐alpha reductase inhibitors showed stronger associations with low‐risk PC than high‐risk PC, though less so for screen‐detected cancers. These suggest that risk factors for low‐risk PC differ from those for high‐risk PC, with determinants of low‐risk PC being more closely related to medical service use.

## What's New?

Overdiagnosis of low‐risk prostate cancer remains an important downside of prostate‐specific antigen‐based screening. This retrospective cohort study of 80,144 men with a median follow‐up of 18.0 years shows that risk factors for low‐risk prostate cancer differ significantly from those for high‐risk prostate cancer. Medical service use was a key determinant for low‐risk cases, while high‐risk cases increased with age. The differences in risk determinants suggest that low‐ and high risk prostate cancers may be regarded as distinct disease entities. The findings can also serve to inform men about the pros and cons of screening and hence facilitate patient decision‐making.

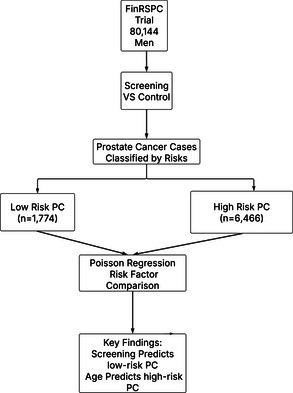

**Abbreviations**
5‐ARI: 5‐alpha reductase inhibitor
CA: control arm
EAU: European Association of Urology
FinRSPC: Finnish Randomized Study of Screening for Prostate Cancer
IR: incidence rate
PC: prostate cancer
PSA: prostate‐specific antigen
SA: screening arm

## 1 INTRODUCTION

Cancer screening is aimed at reducing adverse outcomes of the target disease, most importantly cancer mortality. Systematic testing of asymptomatic people has the potential to detect cancers earlier, at a curable stage and such stage shift is a requirement for effective screening. However, some of the early lesions might not progress to a clinical stage due to either indolent nature of the tumor, or intercurrent death from other causes during the lead‐time gained by screening.

Overdiagnosis or detection of low‐risk prostate cancer (PC) is the most important adverse effects of prostate‐specific antigen (PSA) based PC screening.^1^ Up to half of all screen‐detected cases have been estimated to represent overdiagnosis and screening trials have shown excess incidence amounting to 35–60 excess cases per 1000 men from repeated screening, which should be balanced against 3 averted PC deaths per 1000 men at 16 years.^2^ Most men diagnosed with low‐risk PC do not benefit from early detection yet may be actively treated and suffer the harms of overtreatment.^3^, ^4^ An earlier analysis has already evaluated cancer detection by number of screening rounds attended, though with a shorter follow‐up and different classification of the outcome.^5^

There is currently a debate concerning the terminology of low‐risk PC, as some argue it should not be regarded as a malignant disease^6^, ^7^ Hardly any evidence exists, however, on the factors predicting the diagnosis of low‐risk PC as distinct from clinically relevant PC. In this study, we evaluate the determinants of low‐risk PC within a PSA screening trial and compare the risk factors with those for high‐risk PC.

## 2 PATIENTS AND METHODS

### 2.1 Study design and participants

We investigate the predictors for low‐risk and high‐risk PC in a retrospective cohort study using data from the FinRSPC trial. The FinRSPC trial population originally included 80,458 men born in 1929–1944 recruited during 1996–1999 at ages 55, 59, 63, and 67 years. Of them, 32,000 were randomized to screening up to three times at four‐year intervals during 1996–2007. A total of 314 men were excluded from the screening arm due to PCa diagnosis, death, or emigration from the study area that had occurred prior to randomization, leaving 80,144 men. The details of the FinRSPC study have been published earlier.^8^, ^9^

All PC cases in both arms were identified from the Finnish Cancer Registry^10^ with record linkage based on the unique personal identification numbers.

Clinical details including PSA, Gleason grade, and TNM stage, as well as treatment and subsequent recurrence (including biochemical relapses (BCR)) were abstracted from the medical records or hospital databases. Three experienced uropathologists re‐evaluated Gleason for 1107 cases diagnosed before 2002, using the revised 2005 criteria. This was not done for 696 low‐risk cases diagnosed 2003–2004 (with the pre‐2005 Gleason grading) and those cases were excluded from the analysis. Self‐reported family history was obtained from the men attending the screening and updated at each screening round. Men with an affected brother or father were classified as having a positive family history.

Information on the use of 5‐alpha‐reductase inhibitor (5‐ARI, finasteride, or dutasteride) medication, shown to lower PSA and reduce PC risk in randomized trials,^11^ was obtained from the nationwide prescription database maintained by the Social Insurance Institution. In the analysis, it was classified as any vs. no use during the follow‐up (preceding an eventual PC diagnosis).

Dates and causes of death were obtained from Statistics Finland and those with PC (C61 in ICD‐10) as the underlying cause of death were regarded as PC deaths. Follow‐up started at randomization and ended at PC diagnosis, death, emigration, or the closing date of follow‐up, whichever occurred first. High‐risk PC was treated as censoring at the time of diagnosis for analyses of low‐risk PC, and vice versa. Cancer incidence follow‐up was complete until end of 2016 and causes of death were obtained until the end of 2020.

### 2.2 Statistical analysis

Age‐adjusted incidence rates (IR) (number of cases per 1000 person‐years) of low‐risk PC and high‐risk PC were calculated with follow‐up starting at randomization.

For the analysis, PCs were divided into two categories using the EAU risk groups: low versus intermediate, high, and advanced.^12^ Low‐risk PC was defined according to the EAU risk group classification as cases with a very low risk of progression, Gleason score <7, PSA <10 ng/mL, and stage cT1‐T2a. A total of 1797 such cases were diagnosed. However, 23 low‐risk cases (1.3%) that eventually led to PC death were excluded, leaving 1774 cases for the analysis. PC cases classified as intermediate or high risk, or advanced disease were regarded as high‐risk (*N* = 6466). A total of 1520 cases (15.5%) could not be classified due to missing data on prognostic factors and were excluded. Thus, the primary analysis encompassed 8240 cases in 78,601 men.

A secondary analysis was also conducted where all 561 men with low‐risk PC who subsequently had a BCR or received androgen deprivation therapy (ADT) were also removed, leaving 1213 low‐risk cases.

In an ancillary sensitivity analysis, stage T1a and T1b cases were first excluded as incidental TUR‐related findings, leaving 1461 low‐risk cases. Another ancillary analysis focused on the 1100 screen‐detected cancers only (432 low‐risk and 668 high‐risk PC).

Poisson regression was used to estimate incidence rate ratios (IRR) with 95% confidence intervals (CI) of low‐risk and high‐risk PC. Adjustments for age at entry were used in all analyses. Further adjustment also for the trial arm and 5‐ARI use was evaluated, but it did not change the results and was therefore omitted. No significant interaction was found between trial arm and 5‐ARI use, or trial arm and age. All analyses were conducted using Stata statistical software version 18.0.

Screening participation was analyzed as time‐dependent cumulative number of screening rounds attended, so that all men contributed person‐time (and potentially events) initially to non‐attenders, men participating once then switched to the ‘screened once’ group until their second attendance, and so forth.

## 3 RESULTS

The incidence of low‐risk PC was higher in the SA than the CA (cumulative incidence 2.9% versus 1.8%), while high‐risk PC was slightly more frequent in the CA (8.4% vs. 7.9%). High‐risk PC increased strongly and consistently with age at entry, while indolent PC showed no clear age gradient. Family history of PC and 5‐ARI use were associated with a higher incidence of both low‐risk and high‐risk PC (Table 1).

**TABLE 1:** Number of cases and cumulative incidence (incidence proportion) of low‐risk and high‐risk prostate cancer (PC) with number of men by trial arm, age group, family history, 5‐alpha reductase inhibitor use, and number of screening rounds attended. Low risk defined as low EAU risk group, high‐risk as a higher EAU risk group.

	Low‐risk PC	High‐risk PC	Number of men
	*N* (%)	*N* (%)	
Arm			
Control arm	867 (1.8)	4004 (8.4)	47,522
Screening arm	907 (2.9)	2462 (7.9)	31,079
Age at entry (years)			
55	607 (2.3)	1562 (6.0)	26,043
59	503 (2.4)	1706 (8.3)	20,660
63	392 (2.3)	1598 (9.5)	16,865
67	272 (1.8)	1600 (10.6)	15,033
Family history^a^			
No family history	729 (3.4)	1727 (8.1)	21,359
Affected first degree relative	93 (5.6)	170 (10.3)	1649
Missing	85 (1.1)	565 (7.0)	8071
5‐alpha reductase inhibitor use			
No	1475 (2.1)	5519 (8.0)	69,158
Yes	299 (3.2)	947 (10.0)	9443
Number of screening rounds attended^b^			
Non‐attenders	82 (0.3)	558 (1.8)	31,079
Once	260 (1.1)	731 (3.2)	23,063
Twice	272 (1.5)	597 (3.4)	17,585
Three times	293 (2.9)	576 (5.6)	10,232
Total	1774 (2.3)	6466 (8.2)	78,601

^a^ Screening participants only.

^b^ Screening arm only.

The men in the SA had a higher age‐adjusted IR for low‐risk than those in the CA (IRR 1.62, 95% CI 1.48–1.78) (Table 2). No consistent age pattern was observed for incidence of low‐risk PC, while for high‐risk PC, a clear and consistent increase with age was found. Men with a family history of PC had higher rates of both low‐risk PC and high‐risk PC than those without affected family members, with slightly higher risk estimates for low‐risk PC (IRR 1.61, 95% CI 1.30–2.00 vs. 1.25, 95% CI 1.07–1.46). Men using 5‐ARI had a somewhat higher incidence of low‐risk compared to non‐users, with little difference for high‐risk PC. Incidence of low‐risk PC remained at a constant level across the three screening rounds, whereas the risk increase for high‐risk PC was highest at the first screening attendance.

**TABLE 2:** Age‐adjusted incidence rates (IR) and incidence rate ratios (IRR) of low‐risk and high‐risk prostate cancer with 95% confidence intervals (CI) by trial arm, age group, family history, 5‐alpha reductase inhibitor use, and number of screening rounds attended.

	IR/1000 person‐years (95% CI)		IRR (95% CI)	
	Low‐risk PC	High‐risk PC	Low‐risk PC	High‐risk PC
Arm				
Control arm	1.20 (1.12, 1.28)	5.69 (5.51, 5.87)	1 (reference)	1 (reference)
Screening arm	1.94 (1.82, 2.07)	5.42 (5.20, 5.63)	1.62 (1.48, 1.78)	0.95 (0.91, 1.00)
Age at entry (years)				
55	1.41 (1.30, 1.52)	3.62 (3.44, 3.80)	1 (reference)	1 (reference)
59	1.56 (1.42, 1.70)	5.29 (5.04, 5.54)	1.11 (0.98, 1.25)	1.46 (1.36, 1.56)
63	1.61 (1.45, 1.77)	6.57 (6.24, 6.89)	1.14 (1.01, 1.30)	1.81 (1.69, 1.94)
67	1.41 (1.24, 1.58)	8.28 (7.88, 8.69)	1.00 (0.87, 1.15)	2.29 (2.13, 2.45)
Family history^a^				
No family history	2.17 (2.01, 2.33)	5.21 (4.97, 5.46)	1 (reference)	1 (reference)
Affected first degree relative	3.49 (2.78, 4.20)	6.52 (5.54, 7.51)	1.61 (1.30, 2.00)	1.25 (1.07, 1.46)
5‐alpha reductase inhibitor use				
No	1.43 (1.35, 1.50)	5.58 (5.43, 5.73)	1 (reference)	1 (reference)
Yes	1.90 (1.69, 2.12)	5.60 (5.24, 5.96)	1.34 (1.18, 1.51)	1.00 (0.94, 1.08)
Number of screening rounds attended^b^				
Non‐attenders	1.10 (0.85, 1.34)	5.97 (5.44, 6.50)	1 (reference)	1 (reference)
Once	3.40 (2.93, 3.87)	7.56 (6.94, 8.18)	3.10 (2.41, 4.00)	1.27 (1.13, 1.42)
Twice	3.35 (2.87, 3.83)	5.26 (4.77, 5.76)	3.06 (2.36, 3.96)	0.88 (0.78, 1.00)
Three times	3.59 (3.01, 4.16)	5.78 (5.16, 6.41)	3.27 (2.52, 4.25)	0.97 (0.85, 1.10)

^a^ Screening participants only.

^b^ Screening arm only.

In an analysis of the 1100 screen‐detected prostate cancers alone (432 low‐risk and 668 high‐risk PC), no consistent age pattern was apparent for low‐risk PC, unlike high‐risk PC (Table 3). Family history was associated with a non‐significantly larger risk increase for low‐risk PC than for high‐risk PC (IRR 1.22, 95% 0.87–1.71 vs. 1.09, 95% CI 0.82–1.45). The effect of family history was somewhat smaller compared with the main analysis.

**TABLE 3:** Age‐adjusted incidence rates (IR) and incidence rate ratios (IRR) of low‐risk and high‐risk screen‐detected prostate cancer with 95% confidence intervals (CI) by age group, family history, 5‐alpha reductase inhibitor use, and number of screening rounds attended.

			IR / 1000 person‐years (95% CI)		IRR (95% CI)	
	Low‐risk PC *N* (%)	High‐risk‐PC *N* (%)	Low‐risk PC	High‐risk PC	Low‐risk PC	High‐risk PC
Age at entry (years)						
55	114 (0.5)	120 (0.5)	0.28 (0.23, 0.33)	0.30 (0.24, 0.35)	1 (reference)	1 (reference)
59	134 (0.7)	188 (1.0)	0.45 (0.37, 0.52)	0.63 (0.54, 0.72)	1.59 (1.24, 2.04)	2.12 (1.69, 2.67)
63	118 (0.8)	192 (1.3)	0.52 (0.43, 0.62)	0.85 (0.73, 0.97)	1.86 (1.44, 2.41)	2.88 (2.29, 3.62)
67	66 (0.5)	168 (1.3)	0.37 (0.28, 0.46)	0.95 (0.80, 1.09)	1.32 (0.98, 1.79)	3.20 (2.53, 4.05)
Family history						
No	393 (2.0)	611 (3.1)	1.24 (1.12, 1.36)	1.97 (1.81, 2.12)	1 (reference)	1 (reference)
Positive	37 (2.5)	51 (3.5)	1.51 (1.02, 2.00)	2.14 (1.55, 2.73)	1.22 (0.87, 1.71)	1.09 (0.82, 1.45)
5‐alpha reductase inhibitor use						
No	397 (0.6)	636 (1.0)	0.42 (0.38, 0.46)	0.70 (0.64, 0.75)	1 (reference)	1 (reference)
Yes	35 (0.4)	32 (0.4)	0.23 (0.16, 0.31)	0.20 (0.13, 0.27)	0.56 (0.39, 0.79)	0.29 (0.20, 0.41)

Unlike the main analysis, the findings based on screen‐detected cases only indicate a materially reduced risk of both low‐risk and high‐risk PCa in men using 5‐ARI. This very likely reflects the biological effect of 5‐ARI, while LUTS as the indication for 5‐ARI use often prompts prostate biopsy for differential diagnostics, and this increases PCa detection as reflected in the main results.

The secondary analysis excluding those cases from the low‐risk group that showed evidence for progression after diagnosis gave largely similar results to the main analysis (Table 4). Differences between the age groups were small and inconsistent. The RR associated with a positive family history was higher than in the main analysis. The impact of 5‐ARI use was also accentuated, with a slightly higher effect size. The incidence and IRR of low‐risk PC remained stable across the three screening rounds, consistent with the primary analysis.

**TABLE 4:** Number and incidence proportion (cumulative risk) of low‐risk prostate cancer excluding cases with subsequent androgen deprivation therapy or biochemical recurrence with age‐adjusted incidence rates (IR) per 1000 person‐years (95% confidence interval, CI), and incidence rate ratios (IRR) by trial arm, age group at entry, family history, 5‐alpha reductase inhibitor use, and number of screening rounds attended.

	*N* (%)	IR / 1000 person‐years (95% CI)	IRR (95% CI)
Arm			
Control arm	641 (1.4)	0.92 (0.85, 1.00)	1 (reference)
Screening arm	572 (2.0)	1.27 (1.17, 1.38)	1.38 (1.23, 1.54)
Age at entry (years)			
55	442 (1.8)	1.06 (0.96, 1.16)	1 (reference)
59	341 (1.8)	1.11 (0.99, 1.22)	1.05 (0.91, 1.20)
63	261 (1.7)	1.13 (0.99, 1.26)	1.07 (0.92, 1.24)
67	169 (1.2)	0.93 (0.79, 1.07)	0.88 (0.73, 1.05)
Family history^a^			
No	452 (2.3)	1.39 (1.27, 1.52)	1 (reference)
Positive	57 (3.8)	2.25 (1.66, 2.83)	1.61 (1.22, 2.12)
5‐alpha reductase inhibitor use			
No	978 (1.5)	0.98 (0.92, 1.04)	1 (reference)
Yes	235 (2.7)	1.61 (1.40, 1.81)	1.64 (1.42, 1.90)
Number of screening rounds attended^b^			
Non‐attenders	77 (0.2)	0.76 (0.56, 0.95)	1 (reference)
Once	212 (0.9)	1.72 (1.38, 2.06)	2.28 (1.66, 3.12)
Twice	326 (1.8)	1.70 (1.39, 2.01)	2.25 (1.66, 3.05)
Three times	263 (2.6)	1.93 (1.58, 2.28)	2.55 (1.90, 3.43)

^a^ Screening participants only.

^b^ Screening arm only.

The sensitivity analysis excluding T1a and T1b cases also gave very similar results to the main analysis (Appendix Table S1). The only material difference was that the use of 5‐ARI was no longer associated with low‐risk PC (IRR 1.09, 95% CI 0.94–1.26). Again, there was no material change in low‐risk PC with the number of screening rounds attended.

## 4 DISCUSSION

In this retrospective cohort study, we investigated the incidence and risk factors of low‐risk PC and high‐risk PC in a randomized, PSA‐based screening trial including the post‐intervention phase. Screening attendance was the strongest risk factor for low‐risk PC, followed by family history and allocation to the screening arm. The risk of high‐risk PC was mainly affected by age and, to a lesser extent, family history. Unlike for high‐risk PC, the risk of low‐risk PC showed no consistent gradient with age. Family history and use of 5‐ARI medication showed a stronger association with low‐risk than high‐risk PC. The findings were comparable in secondary analyses excluding cases initially labeled as low‐risk but showing subsequent progression, as well as an analysis excluding T1a and T1b cases. Our results suggest that different factors are driving the detection of low‐risk and high‐risk PC and accord with the notion that these could be regarded as two distinct disease entities.

The finding that detection of low‐risk PC was higher in the screening arm is not novel, as excess incidence has been described both in the ERSPC and other screening trials. Systematic biopsy of all men with elevated PSA results in frequent detection of low‐risk PC and overdiagnosis.^1^, ^2^ If there was no overdiagnosis, the PC incidence in the CA would gradually catch up with the SA with long‐term follow‐up. Within the ERSPC trial, the SA has shown 1.4–1.9‐fold higher PC incidence than the CA at various follow‐up intervals.^13^, ^14^

Our data covers both trial arms, and the screening phase (1996–2007) as well as the post‐intervention follow‐up (2008–2018). Hence, it reflects the full impact of screening, in both short and long term. However, due to a large control arm and increasing incidence with age, most PC cases were detected outside screening. Thus, our results should be broadly generalized, also in a context with opportunistic PSA testing as in our control arm.^8^

The number of screening rounds attended was the strongest predictor for detection of a low‐risk PC. In contrast, the incidence of high‐risk PC was increased only at the first screening attendance after accounting for older age at each subsequent screening round.

Recent analyses of the ERSPC data suggest that the mortality effect of screening is likely to be lower if screening is first started at age 70 years or older, while the age groups with the largest mortality reduction have not been consistent in the three major centers of the ERSPC.^14^, ^15^, ^16^ Major features affecting the mortality impact have been the screening interval and biopsy compliance.^17^, ^18^

Our results provide estimates of probabilities of detecting high‐risk high‐grade cancer versus low‐grade cancer of uncertain clinical importance in screening and outside organized screening programs for several subgroups of the male population, including those with a family history, use of 5‐ARI, and by age group.

Our findings show a larger impact of a positive family history on low‐risk PC than high‐risk PC. Family history is perhaps the best recognized risk factor for PC^19^, ^20^ and men with a family history are recommended for screening as a high‐risk group. Our findings, however, suggest that targeting men with a family history likely increases the detection of both low‐risk and high‐risk PC, with uncertain impact on the balance of benefits and harms. This underscores the importance of evaluating the evidence base for the practice,^21^, ^22^, ^23^ even if it has been widely regarded as intuitively well justified.

For the use of 5‐ARI, a small excess risk was found for mainly for low‐risk PC. This likely reflects a combination of a protective direct biological effect and an indirect effect of the benign prostatic hyperplasia (BPH), for which the medication is prescribed (confounding by indication).^24^ Men with symptomatic BPH are monitored with PSA and likely to undergo PSA testing, with increased potential for detection of low‐risk PC (also at transurethral resection). Interestingly, an analysis restricted to screen‐detected cases showed a substantially reduced risk of both low‐risk and high‐risk PC in men with 5‐ARI use. This supports the interpretation that the biological effect is a risk reduction, but this disappears in clinical practice with PSA testing to exclude PC as the cause of urinary symptoms from BPH.

The true biological effect of 5‐ARI use on PC has been documented in two randomized placebo‐controlled trials (PCPT and REDUCE). Both showed an overall reduction in PCa incidence, but this was due to a reduction in low‐grade cases, while high‐grade tumors were increased. Yet long‐term‐follow‐up of the PCPT trial^25^ has shown a non‐significant decrease in PCa mortality with finasteride, with similar results reported also from large cohort studies of 5‐ARI users.^26^, ^27^

The strengths of the study include the large, population‐based material with comprehensive identification of cases from a nationwide cancer registry.^10^ Our material included more than 8000 PC cases and detailed clinical data from hospital records, also covering disease progression after primary management. Besides prognostic features at diagnosis, we also utilized data on cancer progression and deaths from PC. The context of a randomized screening trial entailed the provision of a systematic screening service with high participation (>70%). Finally, the follow‐up extended to 18 years, covering both the screening period and post‐intervention phase of the study.

In our analysis, we used a refined definition of low‐risk tumor by incorporating also follow‐up data to eliminate cases from the low‐risk category that did not demonstrate a clinical course matching that of a low‐risk tumor despite a low‐grade diagnosis. We therefore used the term low‐risk rather than low‐grade at diagnosis and believe this approach allowed a better distinction of low‐risk cases and reduced misclassification.

We included in the analysis only cases with Gleason <7 scores classified according to the 2005 revision to ensure comparability. Cases diagnosed in 1997–2002 were re‐graded based on a central review by three uropathologists. However, we did not have a centralized grading for the subsequently diagnosed cases. The results were comparable for the cases diagnosed during this period and after 2005. Furthermore, to ensure a valid definition of low‐risk PC, we excluded the cases classified as low risk who eventually died from PC. Further, we used more stringent criteria for secondary analyses, excluding also those cases with evidence for disease progression. The compliance with biopsy after a positive screen in the FinRSPC was very high, 91%.^13^ As a limitation, we did not collect information on all biopsies performed in the trial; only those conducted due to a positive screening result were recorded. Another limitation was the lack of information on LUTS. Our main analyses were based on cases diagnosed during the entire follow‐up period, while a sensitivity analysis was restricted to screen‐detected cases alone. The latter removes the impact of PSA testing (within or outside the trial) and hence represents a biological effect at the time of screening, that is, differences at the time of screening unaffected by differences in PSA testing. The main results provide, however, a better indication of the probability of PCa detection in the real world, where PSA testing is affected by various clinical and socio‐demographic factors. The latter hence are better indicators for informing of their risks in the context of current medical practice.

Our results showing differences in determinants for the two PC subgroups are consistent with the notion that low‐risk cases represent a distinct disease entity different from high‐risk PC.^28^, ^29^ Several studies have suggested that the two phenotypes differ in molecular features.^30^, ^31^

## 5 CONCLUSIONS

Within the 18‐year follow‐up of the FinRSPC trial covering both trial arms, as well as the screening and post‐intervention periods, 30% of the cases were classified as Gleason 6. After the exclusion of cases who subsequently died from PC and those with progression, the strongest predictors of low‐risk PC were randomization to the screening arm and screening attendance, followed by family history and 5‐ARI use. Low‐risk PC did not show a clear gradient with age, unlike high‐risk PC, while only the risk of low‐risk PC increased materially with each screening attendance. Our findings reinforce the importance of balancing the risks of overdiagnosis with the substantial benefits of screening, particularly in reducing PC mortality and improving long‐term clinical outcomes. The differences in risk determinants between the two PC subtypes suggest that they may be distinct disease entities. Our analysis of a very large material with follow‐up spanning up to two decades can serve to inform men about the pros and cons of screening and hence facilitate decision‐making based on individual values and preferences.

## AUTHOR CONTRIBUTIONS

**Uzoamaka E. Okwor:** Conceptualization; methodology; data curation; writing – original draft; writing – review and editing. **Jani Raitanen:** Data curation; writing – review and editing. **Kirsi Talala:** Investigation; writing – review and editing. **Teuvo L. J. Tammela:** Investigation; writing – review and editing; project administration. **Kimmo Taari:** Investigation; writing – review and editing; project administration. **Paula Kujala:** Investigation; writing – review and editing. **Anssi Auvinen:** Conceptualization; supervision; writing – review and editing; writing – original draft; funding acquisition; investigation.

## FUNDING INFORMATION

Supported by grants from the Academy of Finland (grant #260931), Cancer Foundation Finland and State Research Funding administered by Tampere University Hospital.

## CONFLICT OF INTEREST STATEMENT

The authors declare no conflicts of interest relevant to this study.

## ETHICS STATEMENT

This study utilized secondary data obtained from the FinRSPC. The FinRSPC study obtained written informed consent from all screening participants at the time of enrollment and received a statement from the ethical review committees of the Helsinki University Hospital and Tampere University Hospital. Registry data were obtained with permissions from the National Institute of Health and Welfare, Statistics Finland, and the Social Insurance Institution of Finland. The current analysis was conducted using de‐identified data provided by FinRSPC, ensuring participant confidentiality and compliance with ethical guidelines. No additional IRB approval was required for this secondary analysis, as it involved no direct contact with participants and utilized anonymized data.

## Supporting information


Data S1

## Data Availability

The data used in this study were obtained from the Finnish Randomized Study of Screening for Prostate Cancer (FinRSPC), which is part of the European Randomized Study of Screening for Prostate Cancer (ERSPC). To access the data, a request can be made directly to FinRSPC and ERSPC. Further information is available from the corresponding author upon request.
