# Supplementary material for: Risk factors for low‐risk prostate cancer: A retrospective cohort study within the FinRSPC trial
Source: Int J Cancer. 2025 Jul 17;157(10):2033–40. doi: 10.1002/ijc.70026 (PMC12439082; doi:10.1002/ijc.70026)
Supplement: Supplementary file 1 — Data S1 [file IJC-157-2033-s001.pdf]

Supplementary Material for

RISK FACTORS FOR LOW-RISK PROSTATE CANCER: A Retrospective Cohort Study within the FinRSPC Trial

Uzoamaka E. Okwor, Jani Raitanen, Kirsi Talala, Teuvo L.J. Tammela, Kimmo Taari, Paula Kujala, Anssi Auvinen

Appendix Table 1. Age-adjusted incidence rates (IR) and incidence rate ratios (IRR) of low-risk prostate cancer **excluding cancers with stage T1a or T1b**, with 95% confidence intervals (CI) by age group, family history, 5-alpha reductase inhibitor use, and number of screening rounds attended.

	Low-risk PC N (%)	IR / 1000 person-years (95% CI)	IRR (95% CI)
**Arm**			
Control arm	688 (1.5)	0.95 (0.88, 1.02)	1 (reference)
Screening arm	773 (2.5)	1.66 (1.54, 1.78)	1.74 (1.57, 1.93)
**Age at entry (years)**			
55	524 (2.0)	1.22 (1.12, 1.33)	1 (reference)
59	421 (2.1)	1.31 (1.19, 1.44)	1.08 (0.95, 1.22)
63	312 (1.9)	1.29 (1.15, 1.43)	1.06 (0.92, 1.22)
67	204 (1.4)	1.06 (0.92, 1.21)	0.87 (0.74, 1.03)
**Family history***			
No	631 (3.0)	1.88 (1.73, 2.03)	1 (reference)
Positive	75 (4.6)	2.83 (2.19, 3.47)	1.50 (1.18, 1.91)
**5-alpha reductase inhibitor use**			
No	1258 (1.8)	1.22 (1.15, 1.28)	1 (reference)
Yes	203 (2.2)	1.32 (1.14, 1.50)	1.09 (0.94, 1.26)
**Number of screening rounds attended****			
Non-attenders	64 (0.2)	0.92 (0.69, 1.15)	1 (reference)
Once	236 (1.0)	3.20 (2.73, 3.67)	3.48 (2.63, 4.62)
Twice	224 (1.3)	3.13 (2.63, 3.62)	3.40 (2.54, 4.55)
Three times	249 (2.5)	3.60 (2.97, 4.23)	3.91 (2.91, 5.26)

*Screening participants only **Screening arm only
